# Mertansine Inhibits mRNA Expression and Enzyme Activities of Cytochrome P450s and Uridine 5′-Diphospho-Glucuronosyltransferases in Human Hepatocytes and Liver Microsomes

**DOI:** 10.3390/pharmaceutics12030220

**Published:** 2020-03-02

**Authors:** Won-Gu Choi, Ria Park, Dong Kyun Kim, Yongho Shin, Yong-Yeon Cho, Hye Suk Lee

**Affiliations:** Drug Metabolism and Bioanalysis Laboratory, College of Pharmacy, The Catholic University of Korea, Bucheon 14662, Korea

**Keywords:** mertansine, human hepatocytes, cytochrome P450, UDP-glucuronosyltransferases, drug–drug interaction

## Abstract

Mertansine, a tubulin inhibitor, is used as the cytotoxic component of antibody–drug conjugates (ADCs) for cancer therapy. The effects of mertansine on uridine 5′-diphospho-glucuronosyltransferase (UGT) activities in human liver microsomes and its effects on the mRNA expression of cytochrome P450s (CYPs) and UGTs in human hepatocytes were evaluated to assess the potential for drug–drug interactions (DDIs). Mertansine potently inhibited UGT1A1-catalyzed SN-38 glucuronidation, UGT1A3-catalyzed chenodeoxycholic acid 24-acyl-β-glucuronidation, and UGT1A4-catalyzed trifluoperazine *N*-β-d-glucuronidation, with *K_i_* values of 13.5 µM, 4.3 µM, and 21.2 µM, respectively, but no inhibition of UGT1A6, UGT1A9, and UGT2B7 enzyme activities was observed in human liver microsomes. A 48 h treatment of mertansine (1.25–2500 nM) in human hepatocytes resulted in the dose-dependent suppression of mRNA levels of CYP1A2, CYP2B6, CYP3A4, CYP2C8, CYP2C9, CYP2C19, UGT1A1, and UGT1A9, with IC_50_ values of 93.7 ± 109.1, 36.8 ± 18.3, 160.6 ± 167.4, 32.1 ± 14.9, 578.4 ± 452.0, 539.5 ± 233.4, 856.7 ± 781.9, and 54.1 ± 29.1 nM, respectively, and decreased the activities of CYP1A2-mediated phenacetin *O*-deethylase, CYP2B6-mediated bupropion hydroxylase, and CYP3A4-mediated midazolam 1′-hydroxylase. These in vitro DDI potentials of mertansine with CYP1A2, CYP2B6, CYP2C8/9/19, CYP3A4, UGT1A1, and UGT1A9 substrates suggest that it is necessary to carefully characterize the DDI potentials of ADC candidates with mertansine as a payload in the clinic.

## 1. Introduction

**Figure 1:**
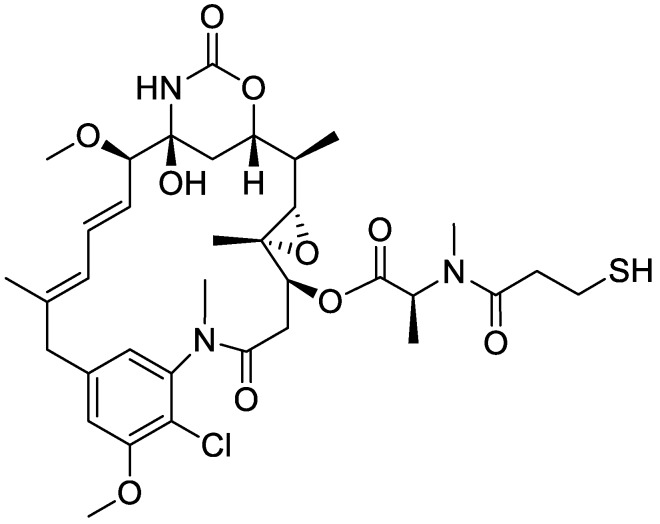
The chemical structure of mertansine.

Maytansine was first isolated in 1972 from the plant *Maytenus* ovatus [1] and showed potent cytotoxic effects in cell-based systems and efficacy in animal tumor models by binding to tubulin and blocking microtubule assembly [1,2,3,4,5]. However, maytansine failed as an anticancer drug in human clinical trials because of its unacceptable systemic toxicity [5,6,7]. Many maytansinoids, chemical derivatives of maytansine, showed higher cytotoxicity—by 100–1000 times—than other tubulin inhibitors, vincristine and vinblastine, in cancer cell lines in vitro [7,8]. The structure–antitumor activity relationship revealed that the ester side chain of maytansine plays an important role in the anti-tumor activity as well as tubulin binding [8]. Maytansinoids with potent cytotoxicity are clinically used and studied as the cytotoxic component of antibody–drug conjugates (ADCs) or aptamer-drug conjugates to reduce side effects and increase treatment effectiveness [7,8,9,10,11,12,13,14,15]. Mertansine (Figure 1, called DM1), a thiol-containing maytansinoid, is attached to a monoclonal antibody through a reaction of the thiol group with a linker to create an ADC. Several ADCs containing mertansine have been developed, including bivatuzumab mertansine, cantuzumab mertansine, lorvotuzummab mertansine, and trastuzumab emtansine (T-DM1, Kadcyla^®^) [6,7,8,9,10,11,12,13,14,15]. T–DM1 is an ADC drug approved in early 2013 for the treatment of human epidermal growth factor receptor 2 (HER2)-positive metastatic breast cancer that combines the biological activity of HER2 antibody (Herceptin or trastuzumab) with the targeted delivery of a potent antimicrotubule agent mertansine to HER2-expressing breast cancer cells [16,17,18,19,20]. A meta-analysis of a total of five randomized clinical trials involving 3,720 patients with HER2-positive metastatic breast cancer revealed that T-DM1 significantly prolonged the progression-free survival and overall survival with tolerated toxicity compared to other anti-HER2 therapies [20]. However, patients who received T-DM1 treatment exhibited a significantly higher risk ratio of hepatotoxicity and thrombocytopenia [20].

Cytochrome P450s (CYPs) and uridine-5′-diphospho-glucuronosyltransferases (UGTs) are critical drug-metabolizing enzymes and are often involved in drug–drug interactions (DDIs) [21,22,23,24,25,26,27]. The in vitro inhibitory and induction potentials of drugs on CYPs and UGTs in human liver microsomes and hepatocytes have been evaluated to help identify clinical DDIs [26].

After an intravenous injection of [^3^H]-mertansine at 0.2 mg/kg in rats, the radioactivity of mertansine was rapidly cleared from the blood and extensively distributed to highly perfused organs such as liver, kidney, spleen, lungs, heart, adrenal, and the gastrointestinal tract with high tissue-to-blood radioactivity ratios (ca. 1~11) for 24 h, declining to minimal levels by 120 h [28]. The majority of dosed mertansine radioactivity was recovered in feces over 120 h, with biliary excretion as the major route (~46% of dosed radioactivity over 72 h), but 5% of dosed radioactivity was recovered in urine over 120 h [28,29]. Mertansine was extensively metabolized to 11 metabolites via *S*-oxidation, hydrolysis, *S*-methylation, and glutathione conjugation [28,30,31]. It competitively inhibited CYP2C8-mediated paclitaxel 6α-hydroxylation and CYP2D6-mediated dextromethorphan *O*-demethylation with *K_i_* values of 11 and 14 µM, respectively, in human liver microsomes; mertansine also inactivated midazolam 1′-hydroxylation in recombinant human CYP3A4 with a *K_i_* of 3.4 µM and a *k*_inact_ of 0.058 min^−1^, but it exhibited no induction potential up to 1 µM [31,32].

Other tubulin inhibitors, such as colchicine and monomethyl auristatin E (MMAE), have been reported to downregulate CYP mRNA expression through the disruption of the microtubulin cellular skeletal structure that is necessary for the proper functioning of nuclear receptor signaling cascades [33,34,35]. However, to our knowledge, no studies have investigated the inhibitory potential of mertansine on UGTs, the second major group of enzymes responsible for drug metabolism [27], in human liver microsomes and the suppression potential of mertansine on mRNA expression or activities of major CYPs and UGTs in human hepatocytes.

The purpose of this study was to investigate the in vitro inhibitory potentials of mertansine on human UGT activities including UGT1A1, UGT1A3, UGT1A4, UGT1A6, UGT1A9, and UGT2B7 in ultrapooled human liver microsomes and to evaluate the effect of mertansine on the mRNA levels of human CYP1A2, CYP2B6, CYP3A4, CYP2C8, CYP2C9, CYP2C19, UGT1A1, UGT1A4, and UGT1A9 in human hepatocytes to assess the potential for mertansine-induced drug interactions.

## 2. Materials and Methods

### 2.1. Materials

Mertansine (96.9% purity) was obtained from BrightGene Biomedical Technology (Jiangsu, China). Acetaminophen, *N*-acetylserotonin, alamethicin, chenodeoxycholic acid, 6-(4-chlorophenyl)imidazo[2,1-b](1,3)thiazole-5-carbaldehyde-*O*-(3,4-dichlorobenzyl)oxime (CITCO), dimethyl sulfoxide (DMSO), l-glutamine, meloxicam, mycophenolic acid, naloxone, naloxone 3-β-d-glucuronide, omeprazole, phenacetin, rifampin, trifluoperazine, Trizma HCl, Trizma Base, uridine 5′-diphosphoglucuronic acid (UDPGA), and William’s Medium E were obtained from Sigma-Aldrich (St. Louis, MO, USA). ^13^C_2_,^15^N-acetaminophen, d_9_-1′-hydroxybufuralol, 1′-hydroxymidazolam, bupropion, hydroxy-bupropion, trypan blue, matrigel, ultrapooled human liver microsomes (150 donors, mixed gender), Biocoat™ Hepatocyte Culture Medium, Biocoat^TM^ Collagen 96-well and 48-well plates, and cryopreserved plateable human hepatocytes (lots 319, 53-year-old male donor; 321, 58-year-old female donor; and 361, 48-year-old female donor) were purchased from Corning Life Sciences (Woburn, MA, USA). *N*-acetylserotonin β-d-glucuronide, chenodeoxycholic acid 24-acyl-β-glucuronide, diclofenac, ketoconazole, mycophenolic acid β-d-glucuronide, propofol β-d-glucuronide, SN-38 glucuronide, and trifluoperazine *N*-β-d-glucuronide were obtained from Toronto Research Chemicals (Toronto, ON, Canada). SN-38 was obtained from Santa Cruz Biotechnology (Dallas, TX, USA). TaqMan^®^ RNA-to-C_T_^TM^ 1-Step Kit, TaqMan^®^ Gene Expression Assays, and gene-specific probes and primers for real-time reverse transcription polymerase chain reaction (RT-PCR) were obtained from Applied Biosystems (Foster City, CA, USA). Midazolam was purchased from Cayman Chemical (Ann Arbor, MI, USA). Cryopreserved hepatocyte recovery medium, cryopreserved hepatocyte plating medium, and fetal bovine serum (FBS) were purchased from Invitrogen (Carlsbad, CA, USA). CellTiter 96^®^ AQueous One Solution Cell Proliferation Assay (MTS) kit was obtained from Promega (Madison, WI, USA). Acetonitrile, methanol, and water (LC-MS grade) were obtained from Fisher Scientific Co. (Fair Lawn, NJ, USA). RNeasy Micro Kit was purchased from QIAZEN (Germantown, MD, USA). All other reagents used were the highest quality available.

### 2.2. Inhibitory Potential of Mertansine on Human Major UGTs in Human Liver Microsomes

The inhibitory potential of mertansine on UGT1A1, UGT1A3, UGT1A4, UGT1A6, UGT1A9, and UGT2B7 activities was evaluated using liquid chromatography–tandem mass spectrometry (LC-MS/MS) with a cocktail of UGT substrates and ultrapooled human liver microsomes [36]. Each incubation mixture was prepared to a final volume of 100 μL as follows: ultrapooled human liver microsomes (0.2 mg/mL), 5 mM UDPGA, 10 mM magnesium chloride, alamethicin (25 μg/mL), 50 mM Tris buffer (pH 7.4), various concentrations of mertansine in methanol (final concentrations of 0.01–50 μM), and the cocktail sets of UGT enzyme-specific substrates. Two cocktail sets were used: set A contained 0.5 μM SN-38 for UGT1A1, 2 μM chenodeoxycholic acid for UGT1A3, and 0.5 μM trifluoperazine for UGT1A4; and set B contained 1 μM N-acetylserotonin for UGT1A6, 0.2 μM mycophenolic acid for UGT1A9, and 1 μM naloxone for UGT2B7. The reactions were initiated by adding UDPGA and incubated in a shaking water bath for 60 min at 37 °C. Reactions were terminated by adding 50 μL of ice-cold acetonitrile containing internal standards (IS, propofol glucuronide for set A and meloxicam for set B). Incubation mixtures were centrifuged at 13,000 g for 8 min at 4 °C. Next, 50 μL of each supernatant of sets A and B was mixed, and aliquots (5 μL) were analyzed using LC-MS/MS. All assays were performed in triplicate and average values were used in the analysis.

The LC-MS/MS system was comprised of an Agilent 6495 triple quadrupole mass spectrometer coupled with an Agilent 1290 Infinity system (Agilent Technologies, Wilmington, DE, USA). The column and autosampler temperatures were set to 40 °C and 4 °C, respectively. Six glucuronide metabolites and two ISs were simultaneously separated using an Atlantis dC_18_ system (3 μm, 2.1 mm i.d. ×100 mm, Waters Technologies, Milford, MA, USA) with a gradient elution of 5% acetonitrile in 0.1% formic acid (MP A) and 95% acetonitrile in 0.1% formic acid (MP B) at a flow rate of 0.3 mL/min. Separation was achieved using the following sequence: 10% MP B for 1 min, 10% to 60% MP B for 1 min, 60% to 95% MP B for 1 min, 95% MP B for 2 min, 95% to 10% MP B for 0.1 min, and 10% MP B for 2.9 min. The electrospray ionization (ESI) source settings in both positive and negative ion modes were as follows: gas temperature, 200 °C; gas flow, 14 L/min; nebulizer, 40 psi; sheath gas temperature, 380 °C; sheath gas flow, 11 L/min; capillary voltage, 4500 V; and nozzle voltage, 500 V. Each metabolite was quantified via selected reaction monitoring in the negative ion mode (chenodeoxycholic acid 24-acyl-β-glucuronide, *m/z* 567.1 to 391.2; mycophenolic acid β-d-glucuronide, *m/z* 495.0 to 319.0; propofol glucuronide (IS), *m/z* 353.0 to 177.0) and in the positive ion mode (SN-38 glucuronide, *m/z* 568.9 to 392.9; trifluoperazine *N*-β-d-glucuronide, *m/z* 583.9 to 407.9; *N*-acetylserotonin β-d-glucuronide, *m/z* 394.0 to 219.0; naloxone 3-β-d-glucuronide, *m/z* 503.9 to 309.9; meloxicam (IS), *m/z* 351.9 to 115.0). Data were processed using MassHunter software (Version B.07.00, Agilent Technologies, Wilmington, DE, USA).

### 2.3. Kinetic Analysis for the Inhibition of UGT1A1, UGT1A3, and UGT1A4 by Mertansine

Kinetic analysis was conducted to determine the *K_i_* values and inhibition mode of mertansine for UGT1A1, UGT1A3, and UGT1A4 enzymes. Human liver microsomes (0.1 mg/mL) were incubated with various concentrations of SN-38 (0.2–2 μM) for UGT1A1, chenodeoxycholic acid (0.5–5 μM) for UGT1A3 or trifluoperazine (0.2–2 μM) for UGT1A4, 5 mM UDPGA, 25 μg/mL alamethicin, 10 mM MgCl_2_, and various concentrations of mertansine (2.5, 5, 10, 20 μM for UGT1A1; 1, 2, 5, 10, 20 μM for UGT1A3; and 2.5, 5, 10, 20, 40 μM for UGT1A4) in 50 mM Tris buffer (pH 7.4) to a total incubation volume of 100 μL. Reactions were initiated by addition of UDPGA at 37 °C and stopped after 30 min by placing the incubation tubes on ice and adding 100 μL of internal standard (500 ng/mL meloxicam for SN-38 glucuronide and trifluoperazine *N*-β-d-glucuronide or propofol glucuronide for chenodeoxycholic acid 24-acyl-β-glucuronide) in ice-cold acetonitrile. The incubation mixtures were then centrifuged at 13,000 g for 4 min, and 50 μL of the supernatant was diluted with 50 μL of water. Aliquots (5 μL) were then analyzed using LC-MS/MS.

### 2.4. Induction of Mertansine on Human Major CYPs and UGTs in Human Hepatocytes 

Plateable cryopreserved human hepatocytes (lots 319, 321, and 361) were thawed in cryopreserved hepatocyte recovery medium according to the manufacturer’s protocol. 

#### 2.4.1. Cytotoxicity of Mertansine in Human Hepatocytes

To estimate the cytotoxicity of mertansine, viable hepatocytes (lot 319) cells were seeded in a collagen type 1 precoated 96-well plate in 100 μL of hepatocyte plating medium (6 × 10^4^ cells/well) and incubated for 4 h at 37 °C in 5% CO_2_. Next, the plating medium was removed, and a matrigel medium containing 0.25 mg/mL of Matrigel™ matrix was applied to each cell prior to incubation for 24 h at 37 °C in 5% CO_2_. The hepatocytes were incubated with 0.0125, 0.0625, 0.125, 0.250, 0.625, 1.25, 2.5, and 6.25 μM mertansine in triplicate for 48 h at 37 °C in 5% CO_2_. The medium was exchanged with fresh medium containing mertansine every 24 h. Then, 20 μL of MTS solution was added to each well and the plate was incubated for 1 h at 37 °C in 5% CO_2_. The absorbance of the reaction mixture was measured at 492 nm.

#### 2.4.2. Treatment of Mertansine in Human Hepatocytes

To evaluate the induction effect of mertansine on drug-metabolizing enzymes, three different cryopreserved human hepatocytes (lots 319, 321, and 361) were thawed in cryopreserved hepatocyte recovery medium, and viable cells were seeded in collagen type 1 precoated 48-well plates in 250 μL of hepatocyte plating medium (6 × 10^5^ cells/well) and incubated for 4 h at 37 °C in 5% CO_2_. Next, the plating medium was removed and replaced with matrigel medium containing 0.25 mg/mL of Matrigel™ matrix prior to incubation at 37 °C for 24 h. The hepatocytes were incubated with 1.25, 12.5, 125, 625, 1250, and 2500 nM mertansine, vehicle (0.1% DMSO in hepatocyte culture media), and prototypical inducers including 50 μM omeprazole, 10 nM CITCO, and 10 μM rifampicin in triplicate. Samples were incubated for 48 h at 37 °C in 5% CO_2_, and the medium was exchanged with 250 μL of fresh medium containing drugs or the vehicle every 24 h.

#### 2.4.3. CYP1A2, CYP2B6, and CYP3A4 Activity Measurement

The effects of mertansine on CYP1A2, CYP2B6, and CYP3A4 activities were evaluated. Plates were prepared with a vehicle, omeprazole, CITCO, rifampin, and mertansine, and incubated for 48 h. Next, 150 µL of a CYP cocktail solution containing 40 µM phenacetin (CYP1A2 substrate), 20 µM bupropion (CYP2B6 substrate), and 20 µM midazolam (CYP3A4 substrate) in William’s E buffer was added to each well and incubated for 30 min, and then 100 µL aliquots of the incubate from each well were stored at −80 °C until LC-MS/MS analysis. ^13^C_2_,^15^N-acetaminophen (0.1 µg/mL, IS for acetaminophen), and d_9_-1′-hydroxybufuralol (0.01 µg/mL, IS for hydroxybupropion and 1′-hydroxymidazolam) in methanol were added to 50 µL of the medium obtained from each well. Mixtures were vortexed for 2 min and then centrifuged at 13,000 g for 4 min at 4 °C. The supernatant (40 µL) was diluted with 60 µL of deionized water and then mixed for 2 min by vortexing. An aliquot (5 µL) was analyzed using LC-MS/MS [37], and CYP1A2, CYP2B6, and CYP3A4 enzyme activities were expressed as formation rates (pmol/million cells/min).

#### 2.4.4. RNA Purification and RT-PCR Analysis

At the end point of the experiment, total RNA was immediately isolated using an RNeasy Micro Kit, and RNA concentration and purity were determined using an absorbance test at 260 nm/280 nm using a NanoVue Plus spectrophotometer (GE Healthcare Bio-Sciences Corp., Piscataway, NJ, USA). Samples were stored at −80 °C until RT-PCR analysis.

RT-PCR analysis was performed using an RT-PCR detection system (Bio-Rad, Hercules, CA, USA) with a TaqMan^®^ RNA-to-CT™ 1-Step Kit and TaqMan^®^ Gene Expression Assay Kits (CYP1A2, Hs01070369_m1; CYP2B6, Hs03044634_m1; CYP3A4, Hs00430021_m1; CYP2C8, Hs00426387_m1; CYP2C9, Hs00426397_m1; CYP2C19, Hs00559368_m1; UGT1A1, Hs02511055_s1; UGT1A4, Hs01655285_s1; UGT1A9, Hs02516855_sH) according to the manufacturer’s protocol. Total RNA (15 ng) in each reaction sample was used for RT-PCR: 25 min for reverse transcription at 48 °C, 15 min for enzyme activation at 95 °C, 44 cycles of denaturation (each 15 s) at 95 °C, and 1 min annealing/extension at 60 °C. The relative threshold cycle (ΔC_t_) values of all samples, including CYP1A2, CYP2B6, CYP3A4, CYP2C8, CYP2C9, CYP2C19, UGT1A1, UGT1A4, and UGT1A9, were normalized to the ΔC_t_ value of glyceraladehyde 3-phosphate dehydrogenase (GAPDH). The relative mRNA abundance was then calculated with the normalized relative C_t_ value (ΔΔC_t_) of each sample using the formula: 2^−(ΔΔC^^t)^. 

### 2.5. Data Analysis

The percentage changes in enzymatic activities were calculated as (CYP activity with test compound treatment/CYP activity with vehicle control treatment) × 100. The IC_50_ (the concentration of the inhibitor needed for half-maximal inhibition) values were calculated using SigmaPlot ver. 12.5 (Systat Software, Inc.; San Jose, CA, USA). *K*_i_ (the inhibition constant) and the inhibition mode of UGT1A1, UGT1A3, and UGT1A4 activities were determined using Enzyme Kinetics ver. 1.1 (Systat Software, Inc.). 

## 3. Results

### 3.1. Inhibition of UGT Enzyme Activities by Mertansine in Human Liver Microsomes

**Figure 2:**
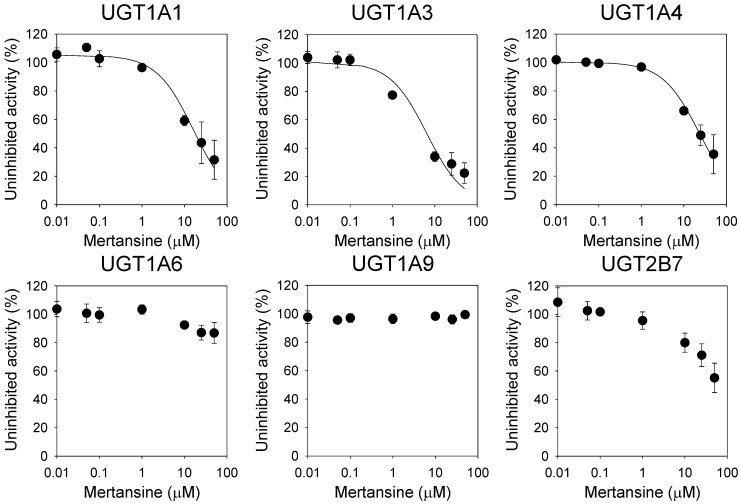
Inhibitory effects of mertansine on six uridine 5′-diphospho-glucuronosyltransferase (UGT) enzyme activities in ultrapooled human liver microsomes. The cocktail UGT substrate concentrations contained 0.5 µM SN-38 for UGT1A1, 2 µM chenodeoxycholic acid for UGT1A3, 0.5 µM trifluoperazine for UGT1A4, 1 µM *N*-acetylserotonin for UGT1A6, 0.2 µM mycophenolic acid for UGT1A9, and 1 µM naloxone for UGT2B7. Data are expressed as means ± SD (*n* = 3).

Mertansine inhibited UGT1A1-catalyzed SN-38 glucuronidation, UGT1A3-catalyzed chenodeoxycholic acid 24-acyl-β-glucuronidation, and UGT1A4-catalyzed trifluoperazine *N*-β-d-glucuronidation with IC_50_ values of 16.2 µM, 6.4 µM, and 23.3 µM, respectively, but negligibly inhibited UGT1A6-catalyzed *N*-acetylserotonin β-d-glucuronidation, UGT1A9-catalyzed mycophenolic acid β-d-glucuronidation, and UGT2B7-catalyzed naloxone 3-β-d-glucuronidation in human liver microsomes at 50 µM (Figure 2, Table 1).

**Figure 3:**
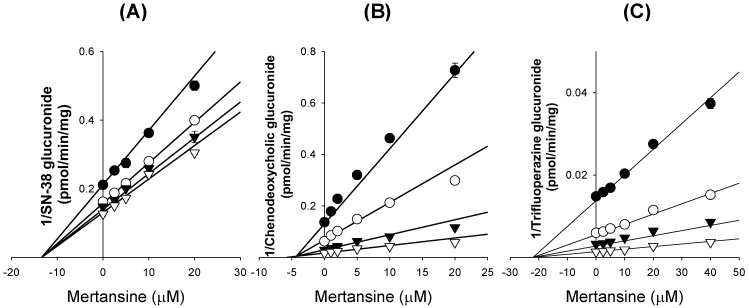
Dixon plots for the inhibitory effects of mertansine on (**A**) UGT1A1-catalyzed SN-38 glucuronidation, (**B**) UGT1A3-catalyzed chenodeoxycholic acid 24-acyl glucuronidation, and (**C**) UGT1A4-catalyzed trifluoperazine *N*-β-d-glucuronidation in ultrapooled human liver microsomes. Several substrate concentrations were evaluated: (**A**) SN-38; 0.2 μM (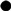); 0.5 μM (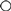); 1 μM (▼); 2 μM (▽); (**B**) chenodeoxycholic acid; 0.5 μM (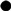); 1 μM (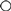); 2 μM (▼); 5 μM (▽); and (**C**) trifluoperazine; 0.2 μM (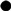); 0.5 μM (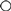); 1 μM (▼); 2 μM (▽). Data are expressed as means ± SD (*n* = 3).

Mertansine noncompetitively inhibited UGT1A1-catalyzed SN-38 glucuronidation with a *K*_i_ value of 13.5 μM, and competitively inhibited UGT1A3-catalyzed chenodeoxycholic acid 24-acyl-glucuronidation and UGT1A4-catalyzed trifluoperazine *N*-β-D-glucuronidation, with *K*_i_ values of 4.3 and 21.2 μM, respectively (Figure 3, Table 1).

### 3.2. Effects of Mertansine on CYP and UGT mRNA Levels in Human Hepatocytes

In the MTS colorimetric assay, mertansine did not cause toxicity in human hepatocytes (lot 319), as the viability of hepatocytes following 48 h mertansine treatment (1.25–6250 nM) was over 96.2%.

The functionality of the hepatocyte was confirmed by the increase of mRNA levels and enzyme activities of CYPs following 48 h treatment with prototypical inducers using RT-PCR and LC-MS/MS, respectively, compared to the vehicle (Table 2). Fifty micromoles of omeprazole, a representative aromatic hydrocarbon receptor inducer (AHR), increased the CYP1A2 mRNA levels by enhancing the AHR binding to the promoter region of CYP1A2 [38] and CYP1A2-mediated phenacetin *O*-deethylase activity by 58.7–299.3 and 11.7–61.8 fold, respectively (Table 2). 10 μM rifampin, a potent pregnane X receptor (PXR) inducer, increased mRNA levels of CYP3A4 by enhancing the PXR binding to the promoter region of CYP3A4 [39] and CYP3A4-mediated midazolam 1′-hydroxylase by 74.0–146.7 and 3.6–9.8 fold, respectively (Table 2). Additionally, 10 nM CITCO increased CYP2B6 mRNA levels and CYP2B6-mediated bupropion hydroxylase activity by 5.6–8.7 and 3.8–15.7 fold, respectively (Table 2), which was mediated by the transcriptional activation by the enhancement of constitutive androstane receptor binding to the promoter region of CYP2B6 [40]. 10 μM rifampin increased mRNA levels of CYP2C8, CYP2C9, CYP2C19, UGT1A1, UGT1A4, and UGT1A9 by 3.7–4.8, 2.9–5.3, 2.0–2.2, 2.5–3.0, 3.9–4.5, and 2.0–2.2 fold, respectively, and 50 μM omeprazole increased the mRNA levels of UGT1A1 and UGT1A4 by 3.9–7.0 and 3.3–4.1 fold, respectively, in three human hepatocytes (Table 2).

**Figure 4:**
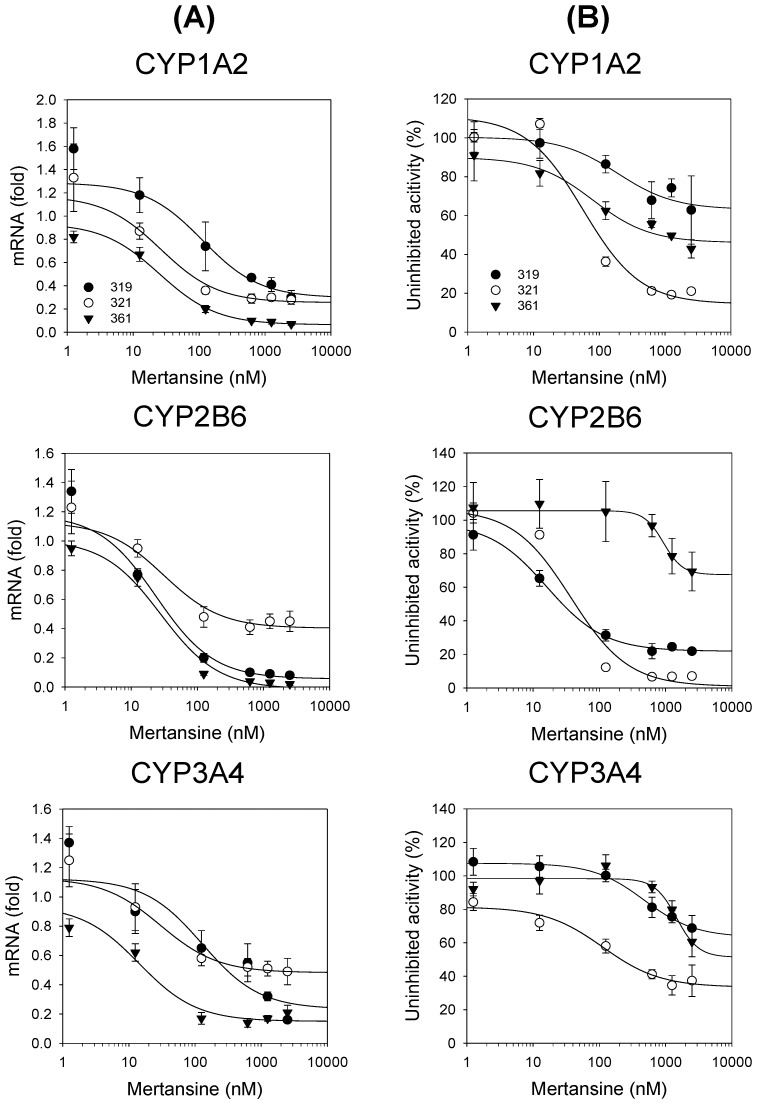
Effects of mertansine on (**A**) the mRNA levels of CYP1A2, CYP2B6, and CYP3A4; and (**B**) the activities of CYP1A2-catalyzed phenacetin *O*-deethylase, CYP2B6-catalyzed bupropion hydroxylase, and CYP3A4-catalyzed midazolam hydroxylase compared to the vehicle (0.1% DMSO) after 48 h mertansine treatment (1.25–2500 nM) in three human hepatocytes: lots 319 (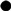), 321 (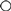), and 361 (▼). Data are expressed as means ± SD (*n* = 3).

Mertansine led to the dose-dependent suppression of mRNA expression of CYP1A2 (from 1.2 to 0.22 fold), CYP2B6 (from 1.2 to 0.18 fold), and CYP3A4 (from 1.1 to 0.29 fold) in three human hepatocytes (Figure 4A). Mertansine decreased the activities of CYP1A2-mediated phenacetin *O*-deethylase by 27.8–79.0%, CYP2B6-mediated bupropion hydroxylase by 23.9–93.1%, and CYP3A4-mediated midazolam 1′-hydroxylase by 30.8–62.7%, compared to the enzyme activities treated with the vehicle in three human hepatocytes (Figure 4B).

**Figure 5:**
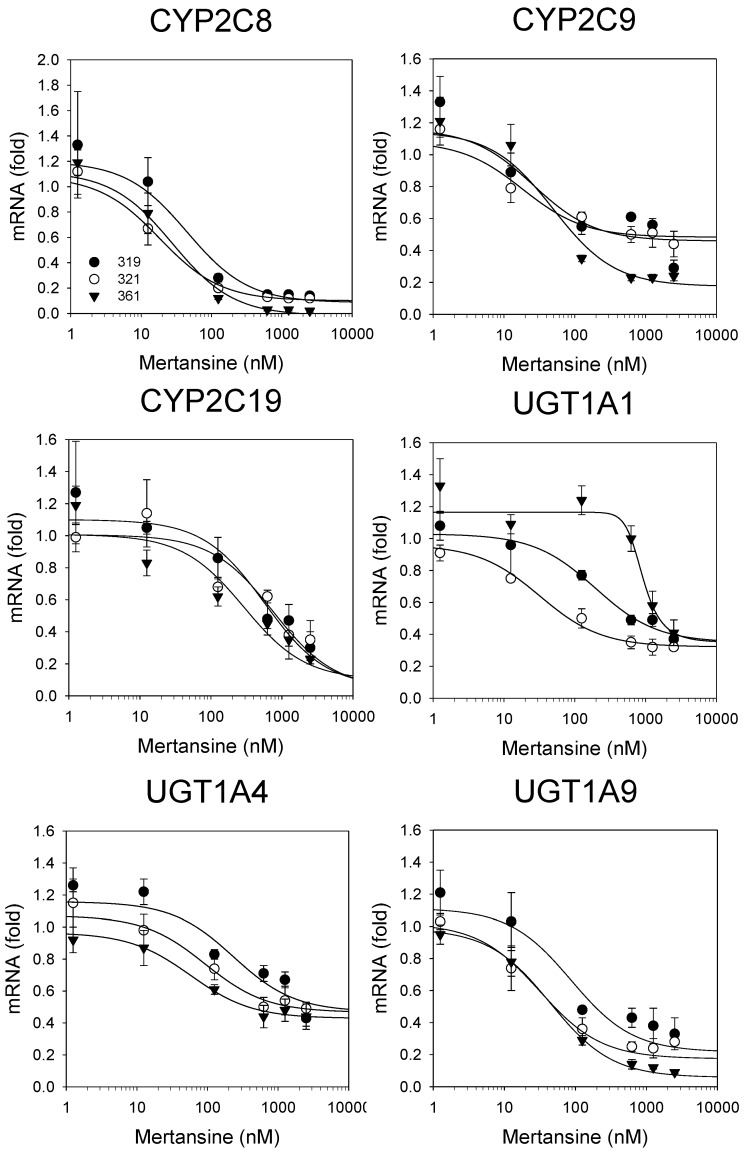
Effects of mertansine on mRNA levels of CYP2C8, CYP2C9, CYP2C19, UGT1A1, UGT1A4, and UGT1A9 after 48 h treatment in three human hepatocytes: lots 319 (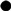), 321 (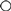), and 361 (▼). Data are expressed as means ± SD (*n* = 3).

Mertansine dose-dependently suppressed the mRNA levels of CYP2C8 (from 1.2 to 0.09 fold), CYP2C9 (from 1.2 to 0.32 fold), CYP2C19 (from 1.3 to 0.23 fold), UGT1A1 (from 1.1 to 0.37 fold), UGT1A4 (from 1.1 to 0.45 fold), and UGT1A9 (from 1.2 to 0.09 fold), in three human hepatocytes (Figure 5). Table 3 lists the IC_50_ values for mertansine on the suppression of mRNA expression of CYPs and UGTs in three human hepatocytes.

## 4. Discussion

In this study, the effects of mertansine on the inhibition of UGT activities in human liver microsomes and its effects on mRNA expression of CYPs and UGTs in human hepatocytes were evaluated to assess the potential for mertansine-induced drug interactions.

Mertansine was a noncompetitive inhibitor of UGT1A1-catalyzed SN-38 glucuronidation with a *K_i_* value of 13.5 μM, and a competitive inhibitor of UGT1A3-catalyzed 24-acyl-β-glucuronidation and UGT1A4-catalyzed trifluoperazine *N*-β-d-glucuronidation with *K_i_* values of 4.3 and 21.2 μM, respectively, in human liver microsomes (Figure 3). These findings suggest the potential for DDIs between mertansine and UGT1A1, UGT1A3, or UGT1A4 substrates when used concomitantly. However, the maximum plasma concentrations of mertansine were 7.2 ± 2.7 nM, with the highest level of 30 nM after the intravenous infusion of 3.6 mg/kg T-DM1 every 3 weeks in HER2-positve breast cancer patients [16,17,18,19]. Therefore, the ratio of maximal unbound plasma concentrations of mertansine to *K_i_* values (0.00004–0.0002) was much lower than the ratio indicating the likelihood of drug interaction (0.1), suggesting that mertansine-induced drug interactions via the inhibition of UGT activity are unlikely during T-DM1 therapies.

In addition, although mertansine is a competitive inhibitor of CYP2C8 and CYP2D6 activities with *K_i_* values of 11 and 14 µM, respectively, and it also irreversibly inhibits CYP3A4 activity with *K_i_* of 3.4 µM and *k*_inact_ of 0.058 min^−1^, mertansine would not cause serious CYP-mediated DDI during the T-DM1 therapies considering the plasma concentrations [32].

The mRNA levels and enzyme activities of CYP1A2, CYP2B6, and CYP3A4 were induced to levels comparable to typical inducers, such as omeprazole, CITCO, and rifampin, following 48 h treatment in three human hepatocytes (Table 2), indicating that the induction system used herein was reliable. The induction effects of mertansine on CYPs and UGTs were assessed using therapeutic to clinically non-achievable high concentrations (1.25–2500 nM) in three human hepatocytes. Mertansine dose-dependently suppressed the mRNA expression of CYP1A2, CYP2B6, and CYP3A4 with IC_50_ values of 93.7 ± 109.1, 36.8 ± 18.3, and 160.6 ± 167.4 nM, respectively, in thee human hepatocytes (Figure 4A, Table 3). Additionally, mertansine decreased the activities of CYP1A2-mediated phenacetin *O*-deethylase, CYP2B6-mediated bupropion hydroxylase, and CYP3A4-mediated midazolam 1′-hydroxylase by mean values of 48.3%, 63.5%, and 39.7%, respectively, at the highest concentration (2500 nM) in three human hepatocytes (Figure 4B). Mertansine dose-dependently suppressed the mRNA expression of CYP2C8, CYP2C9, CYP2C19, UGT1A1, and UGT1A9 mRNA levels with IC_50_ values of 32.1 ± 14.9, 578.4 ± 452.0, 539.5 ± 233.4, 856.7 ± 781.9, and 54.1 ± 29.1 nM, respectively, with a little suppression of UGT1A4 mRNA levels (IC_50_ value > 2500 nM) (Figure 5, Table 3). These suppressions were not likely to be due to cytotoxic effects because the viability of hepatocytes was not affected by mertansine treatment (1.25–6250 nM). The suppression of CYP mRNA by mertansine was similar to those of other tubulin inhibitors, such as MMAE and colchicine [33,34,35]. In previous studies, 100 and 1000 nM MMAE treatment suppressed CYP1A2, CYP2B6, and CYP3A4 mRNA expression by 61–90% and 95–97%, respectively, and decreased CYP activities by 40–71% and 45–81%, respectively, in three human hepatocytes [32]. These findings support the idea that the suppression of CYP and UGT mRNA levels by mertansine may result from the disruption of cytoskeletal structures formed by microtubule networks, which are important for the functioning of the nuclear receptor signaling cascade [33,34,35,41]. These in vitro results suggest the clinical evaluation of the DDI potential of mertansine with CYP1A2, CYP2B6, CYP3A4, CYP2C8/9/19, UGT1A1, and UGT1A9 substrates.

Several ADCs with mertansine as a payload have been under clinical trials since the approval of T-DM1 [7,10,11,12,13,14]. Liver is the major organ for the distribution and metabolism of antibody maytansinoid conjugates and its catabolites, and the hepatic concentrations of mertansine or ravtansine therefore depend on the catabolism of ADC within the liver [7,18,29,42]. The extensive tissue distribution of mertansine after the administration of mertansine itself in rats led to higher hepatic levels of mertansine compared to plasma levels [28]. Although the maximal plasma concentration of the catabolite mertansine is low (≤7.2 ± 2.7 nM) in T-DM1 treated cancer patients [16,17,18,19], a clinical evaluation of DDIs regarding the reduced mRNA levels by repeated treatment of T-DM1 and the CYP1A2, CYP2B6, CYP2C8/9/19, CYP3A4, UGT1A1, and UGT1A9 substrates may be necessary on the basis of these in vitro findings.

## 5. Conclusions

Mertansine inhibited UGT1A1, UGT1A3, and UGT1A4 enzyme activities in human liver microsomes and dose-dependently suppressed the mRNA levels of CYP1A2, CYP2B6, CYP3A4, CYP2C8, CYP2C9, CYP2C19, UGT1A1, UGT1A4, and UGT1A9 after 48 h treatment of 1.25–2500 nM mertansine in three human hepatocytes. Additionally, mertansine treatment resulted in the decrease of CYP1A2, CYP2B6, and CYP3A4 enzyme activities. These in vitro DDI potentials of mertansine with substrate drugs for major CYPs and UGTs enzymes indicate that the evaluation of the DDI potentials of ADC candidates with mertansine as a payload is necessary.

## Figures and Tables

**Table 1:** Inhibitory potentials of mertansine on six UGT enzyme activities in ultrapooled human liver microsomes.

UGTs	Enzyme Activities	IC_50_ (µM)	*K_i_* (µM)	Inhibition Mode
1A1	SN-38 glucuronidation	16.2	13.5	Noncompetitive
1A3	Chenodeoxycholic acid 24-acyl-β-glucuronidation	6.4	4.3	Competitive
1A4	Trifluoperazine *N*-β-d-glucuronidation	23.3	21.2	Competitive
1A6	N-acetylserotonin β-d-glucuronidation	No inhibition	-	-
1A9	Mycophenolic acid β-d-glucuronidation	No inhibition	-	-
2B7	Naloxone 3-β-d-glucuronidation	No inhibition	-	-

-: not assayed.

**Table 2:** Effects of prototypical inducers such as omeprazole, 6-(4-chlorophenyl)imidazo[2,1-b](1,3)thiazole-5-carbaldehyde-*O*-(3,4-dichlorobenzyl)oxime (CITCO), and rifampicin on the mRNA expression of cytochrome p450s (CYPs) and UGTs and the enzyme activities of CYP1A2, CYP2B6, and CYP3A4 after 48 h treatment in three human hepatocytes (lots 319, 321, and 361). Data are expressed as means ± SD (*n* = 3).

Enzymes	mRNA (Fold Change)			Enzyme Activities (pmol/10^6^ Cells/min)		
	Lot 319	Lot 321	Lot 361	Lot 319	Lot 321	Lot 361
**Omeprazole 50 μM**						
Vehicle control	1.0	1.0	1.0	1.6 ± 0.40 ^a^	3.10 ± 0.26 ^a^	1.3 ± 0.16 ^a^
CYP1A2	132.4 ± 0.22	58.7 ± 4.7	299.3 ± 49.0	18.5 ± 3.3 ^a^	89.45 ± 4.52 ^a^	81.0 ± 0.53 ^a^
UGT1A1	6.97 ± 0.94	4.05 ± 0.53	3.9 ± 0.24	-	-	-
UGT1A4	3.31 ± 0.35	4.13 ± 0.66	3.7 ± 0.62	-	-	-
**CITCO 10 nM**						
Vehicle control	1.0	1.0	1.0	0.68 ± 0.23 ^b^	0.72 ± 0.10 ^b^	3.43 ± 0.48 ^b^
CYP2B6	7.4 ± 1.9	5.6 ± 1.6	8.7 ± 0.32	9.2 ± 0.28 ^b^	11.3 ± 0.49 ^b^	12.9 ± 0.51 ^b^
**Rifampin 10 μM**						
Vehicle control	1.00	1.00	1.00	16.6 ± 3.78 ^c^	4.3 ± 0.09 ^c^	7.1 ± 0.67 ^c^
CYP3A4	74.0 ± 10.3	146.7 ± 24.9	129.8 ± 0.5	74.9 ± 3.36 ^c^	42.0 ± 9.8 ^c^	25.6 ± 1.0 ^c^
CYP2C8	3.7 ± 0.57	4.8 ± 0.84	4.0 ± 0.16	-	-	-
CYP2C9	5.3 ± 0.02	2.9 ± 0.11	3.7 ± 0.36	-	-	-
CYP2C19	2.2 ± 0.09	2.0 ± 0.18	2.1 ± 0.25	-	-	-
UGT1A1	2.9 ± 0.13	3.0 ± 0.11	2.5 ± 0.37	-	-	-
UGT1A4	4.0 ± 0.22	4.5 ± 0.59	4.5 ± 0.87	-	-	-
UGT1A9	2.0 ± 0.23	2.2 ± 0.31	2.1 ± 0.23	-	-	-

-: not assayed; vehicle control: 0.1% DMSO treatment; ^a^: CYP1A2-catalyzed phenacetin *O*-deethylase activity; ^b^: CYP2B6-catalyzed bupropion 1′-hydroxylase activity; ^c^: CYP3A4-catalyzed midazolam 1′-hydroxylase activity.

**Table 3:** IC_50_ values for mertansine on the suppression of mRNA expression of CYPs and UGTs after 48 h mertansine treatment (1.25–2500 nM) in three human hepatocytes (lots 319, 321, and 361).

Enzymes	IC_50_ (nM)			
	Lot 319	Lot 321	Lot 361	Mean ± SD
CYP1A2	219.7	34.3	27.3	93.7 ± 109.1
CYP2B6	25.0	57.8	27.6	36.8 ± 18.3
CYP3A4	344.5	120.5	16.8	160.6 ± 167.4
CYP2C8	48.7	20.0	27.5	32.1 ± 14.9
CYP2C9	788.2	887.5	59.6	578.4 ± 452.0
CYP2C19	573.5	754.1	291.0	539.5 ± 233.4
UGT1A1	784.5	113.4	1672.1	856.7 ± 781.9
UGT1A4	>2500	>2500	>2500	>2500
UGT1A9	86.9	31.4	44.1	54.1 ± 29.1
